# Postmenopausal Shrinkage of Uterine Myomas: A Retrospective Study of 97 Cases Monitored Annually for 10 Years

**DOI:** 10.7759/cureus.70656

**Published:** 2024-10-01

**Authors:** Kenta Oue, Tomoyuki Ichimura, Makoto Murakami, Makiko Matsuda, Naoki Kawamura, Takeshi Fukuda, Toshiyuki Sumi

**Affiliations:** 1 Obstetrics and Gynecology, Osaka City University Graduate School of Medicine, Osaka, JPN; 2 Gynecology, Osaka City General Hospital, Osaka, JPN; 3 Gynecology, Izumi City General Hospital, Osaka, JPN; 4 Gynecology, Nishi-Umeda City Clinic, Osaka, JPN; 5 Obstetrics and Gynecology, Osaka Metropolitan University School of Medicine, Osaka, JPN

**Keywords:** long-term follow-up, linear mixed model, number of nodules, body mass index, postmenopausal shrinkage, uterine myoma

## Abstract

Introduction and aim

Both patients and gynecologists are concerned about how much and how quickly myomas shrink after menopause. This study aimed to elucidate clinical findings that may be associated with postmenopausal shrinkage of uterine myomas.

Materials and methods

This study included 97 patients who underwent menopause by August 2012, had myoma nodules with the longest diameter between 50 mm and 160 mm, and visited our specialized myoma clinic annually for at least 10 years after menopause. They underwent transabdominal ultrasonography at least once per year. An experienced gynecologist measured the longest diameter of myoma nodules with a maximum diameter between 50 mm and 160 mm. The shrinkage rate of myoma diameters after menopause compared to premenopausal diameters was calculated each year for 10 years. The shrinkage rate of the longest diameter of the largest nodule 10 years after menopause (10-year shrinkage rate) and its relationship with clinical findings (the age at menopause, parity, body mass index {BMI}, number of nodules, MRI findings on T2-weighted image, location of the nodule, and longest diameter of the largest nodule before menopause) were analyzed. Additionally, we examined annual changes in shrinkage rate of myomas over a 10-year period after menopause (annual trend), and the relationship between annual trends and factors such as BMI and the number of nodules.

Results

In this examination of 10-year shrinkage rate, the group with a BMI of less than 25 showed a significantly greater shrinkage rate compared to the group with a BMI of 25 or more (25.0% vs 15.7%, p=0.023). Additionally, the group with a single nodule showed a significantly greater 10-year shrinkage rate compared to the group with four or more nodules (26.3% vs 15.2%, p=0.036). For annual trends, the rate of change in the first two years after menopause was significantly faster compared to the trend from the third to the 10th year (difference in slope: 3.888 points per year, p<0.001). When divided into two groups based on the number of nodules (one or two nodules group and three or more nodules group), the group with one or two nodules showed a significant difference in the shrinkage rate between up to two years after menopause and from the period from the third to the 10th year (difference in slope: 4.590 points per year, p<0.001). However, for the group with three or more nodules, there was no significant difference in the annual trend between the first two years after menopause and the rate from the third to the 10th year (difference in slope: 1.626 points per year, p=0.107).

Conclusion

BMI and the number of myoma nodules were significantly related to the 10-year shrinkage rate. Although myomas shrank significantly faster within the first two years after menopause compared to the later period, the early annual trend did not differ significantly from the trend in the later period when there were multiple nodules with a maximum diameter of 50 mm or more.

## Introduction

Uterine myomas (myomas) are the most common tumors, found in 20-30% of females over the age of 30 years [1]. The symptoms of myomas vary widely depending on their size and location. Symptomatic myomas, particularly those associated with menstrual symptoms, often need surgery. However, in cases of asymptomatic myomas, mild menstrual-related symptoms that can be managed with temporary expedient, or compressive symptoms that are tolerable for the patient, conservative management is often employed even when the myomas are large. Particularly subserosal and intramural myomas, even if they are large, are often managed with observation. The basis for such management is the fact that myomas are benign tumors that naturally shrink after menopause. In this context, both patients and gynecologists are concerned about how much and how quickly myomas shrink after menopause when relatively large myomas are managed conservatively. Despite numerous studies and papers on shrinkage due to pseudomenopause therapy, there are few research and publications on the shrinkage of myomas after menopause [2]. Our group has previously conducted and reported studies on the shrinkage rates of myomas after pseudomenopause therapy and after menopause [3]. In this previous report, the median observation period postmenopause was two years (ranging from one to 5.5 years). In real-world clinical practice, some myomas shrink dramatically within a few years after menopause. On the other hand, some myomas remain almost unchanged in size for several years after menopause. Long-term postmenopausal follow-up is needed to elucidate whether the rate of myoma shrinkage changes thereafter, but there have been no such reports to date. At our hospital's specialized myoma clinic, we have been conducting long-term follow-ups even after menopause. By utilizing these data, we were able to examine the changes in myoma size over a 10-year period after menopause, allowing us to study the long-term size changes of myomas after menopause.

This article was previously presented as a meeting abstract at the 76th Annual Congress of the Japan Society of Obstetrics and Gynecology, held from April 19 to 21, 2024.

## Materials and methods

Subjects

This study included 97 cases of myomas in patients attending our outpatient specialized myoma clinic at Osaka City University Hospital, who experienced menopause before August 2012, had nodules with the longest diameter between 50 mm and 160 mm, and visited the clinic annually for at least 10 years after menopause, undergoing transabdominal ultrasonography at least once per year. Cases with more than 160 mm myoma nodule, unclear margins, history of malignant disease, history of hormone therapy, and uterine artery embolization (UAE) therapy were excluded. This study was approved by the Ethics Committee of Osaka City University (approval number: 2023-004).

Methods

A single experienced gynecologist performed transabdominal ultrasonography using either LOGIQ7 (Chicago, IL: GE HealthCare) or ALOKA ARIETTA 850 (Tokyo, Japan: Fujifilm). The longest diameter of the myoma nodules was measured in the sagittal plane at the point where they were longest without deviating from the body's axis. In cases where multiple nodules had the longest diameters between 50 mm and 160 mm, the nodule with the maximum diameter was targeted. For the measurement of the number of nodules, only those measured in the sagittal plane with a diameter between 50 mm and 160 mm were counted. The shrinkage rate of myoma diameters after menopause compared to premenopausal diameters was calculated for each year. Shrinkage rate at 10th year after menopause (10-year shrinkage rate) and its relationship with clinical findings (age at menopause, parity, body mass index {BMI}, number of nodules, MRI findings on T2-weighted image, location of nodule, and longest diameter of the largest nodule before menopause) were analyzed. BMI was calculated by dividing weight (kg) by the square of height (m), and patients were divided into the two following groups according to BMI: BMI less than 25 and BMI 25 or higher. For the number of nodules, patients were divided into the following four groups: one nodule, two nodules, three nodules, and four or more nodules. MRI findings were categorized into groups with or without high signal intensity compared to uterine myometrium on T2-weighted images. For parity, patients were divided into the following four groups: zero, one, two, and three or more times. We examined annual changes in shrinkage rate of myomas over a 10-year period after menopause (annual trends). We examined whether there were differences in annual trends between the first two years and three to 10 year after menopause. Additionally, we investigated the relationship between annual trends and factors, such as BMI and number of nodules. These data were retrospectively reviewed using our hospital's medical records.

Statistical analysis

For analysis of relationship between 10-year shrinkage rate and clinical findings, Student’s t-tests were conducted for parity, BMI, number of nodules, MRI findings, and nodule location. Spearman’s rank correlation tests were conducted for the longest diameter of the largest nodule before menopause and age at menopause. A stepwise multiple linear regression analysis was also performed for these seven factors. The linear mixed-effects model was employed to determine if there were significant differences in the annual trends over the 10 years and if clinical findings affected the shrinkage speed. Statistical analyses were performed with R version 4.2.3 (Vienna, Austria: R Foundation for Statistical Computing). A p-value of less than 0.05 was considered statistically significant.

## Results

The age at menopause, parity, BMI, number of nodules, MRI findings on T2-weighted images, location of nodules, and the longest diameter of the largest nodule before menopause in the 97 cases who participated in this study are shown in Table 1. The patients’ age at menopause ranged from 45 to 59 years with a median age of 52 years. Their parity ranged from zero to four times with a median age of one time. BMI ranged from 16.2 to 34.6 with a median of 22.4. Among the patients, 50.5% of patients had single myoma nodules, 25.8% had two nodules, 9.3% had three nodules, and 14.4% had four or more nodules. Among the patients, 23.9% of target nodules had a high signal at T2-weighed image. Among the patients, 69.1% of target nodules were intramural, and 30.9% were subserous. The maximum premenopausal diameter measured in US ranged from 53 to 144 mm, with a median of 81 mm.

**Table 1:** Characteristics of all patients (n=97).

Characteristics (n=97)	Values
Age at menopause (years), median (range)	52 (45-59)
Parity, median (range)	1 (0-4)
Body mass index (kg/m^2^), median (range)	22.4 (16.2-34.6)
Number of myoma nodules	
1, n (%)	49 (50.5)
2, n (%)	25 (25.8)
3, n (%)	9 (9.3)
≥4, n (%)	14 (14.4)
MRI high signal at T2, n (%)	21 (23.9)
Site of nodule	
Intramural, n (%)	67 (69.1)
Subserous, n (%)	30 (30.9)
Maximum diameter before menopause (mm), median (range)	81 (53-144)

**Figure 1:**
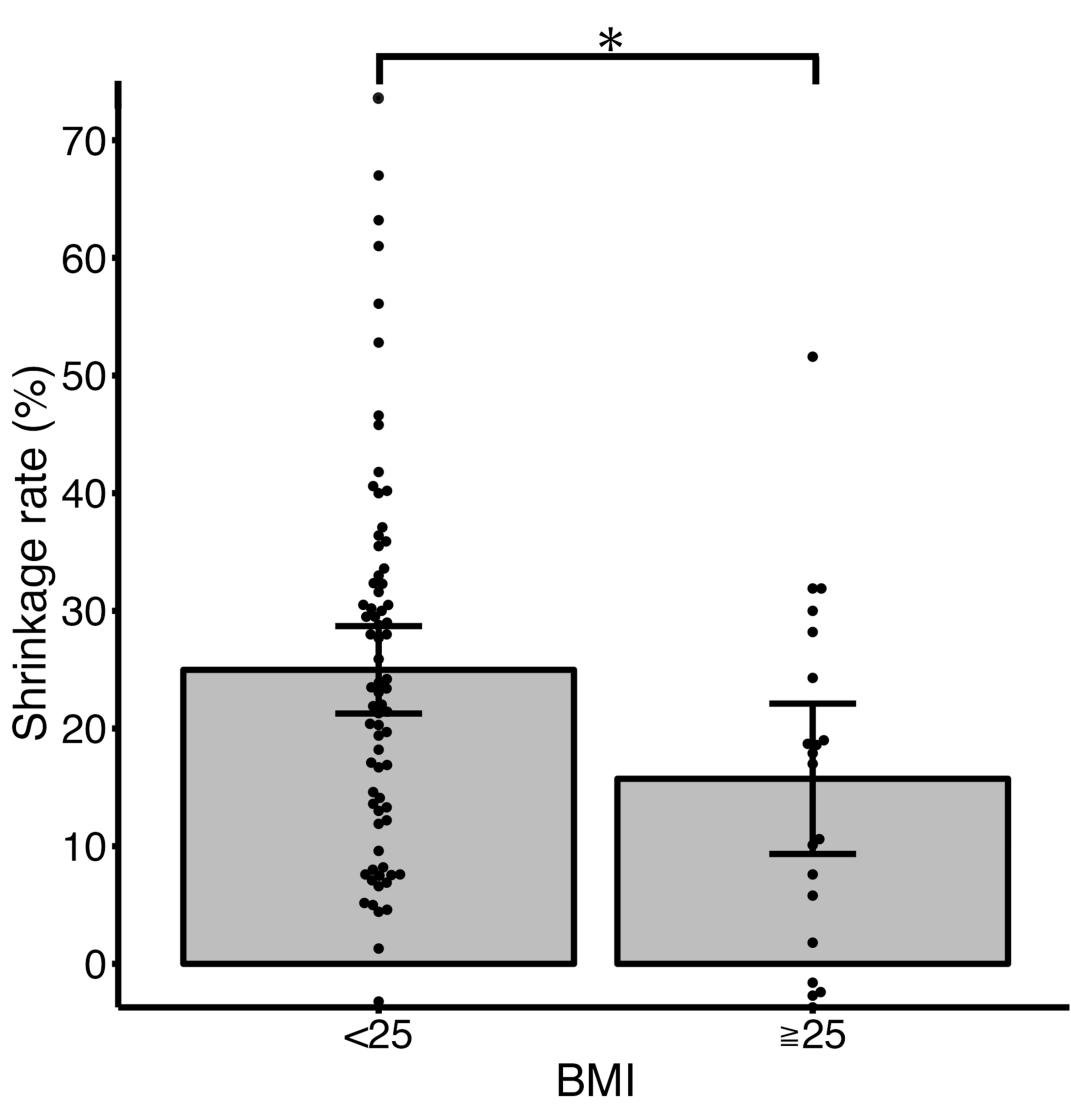
Mean shrinkage rate at 10-year after menopause with 95% confidence interval based on BMI. The BMI <25 group had a significantly higher mean shrinkage rate compared to the BMI ≥25 group (25.0% vs 15.7%). Dots show each patient’s shrinkage rate. Bars show a 95% confidence interval. The asterisk shows a p-value of 0.023.

**Figure 2:**
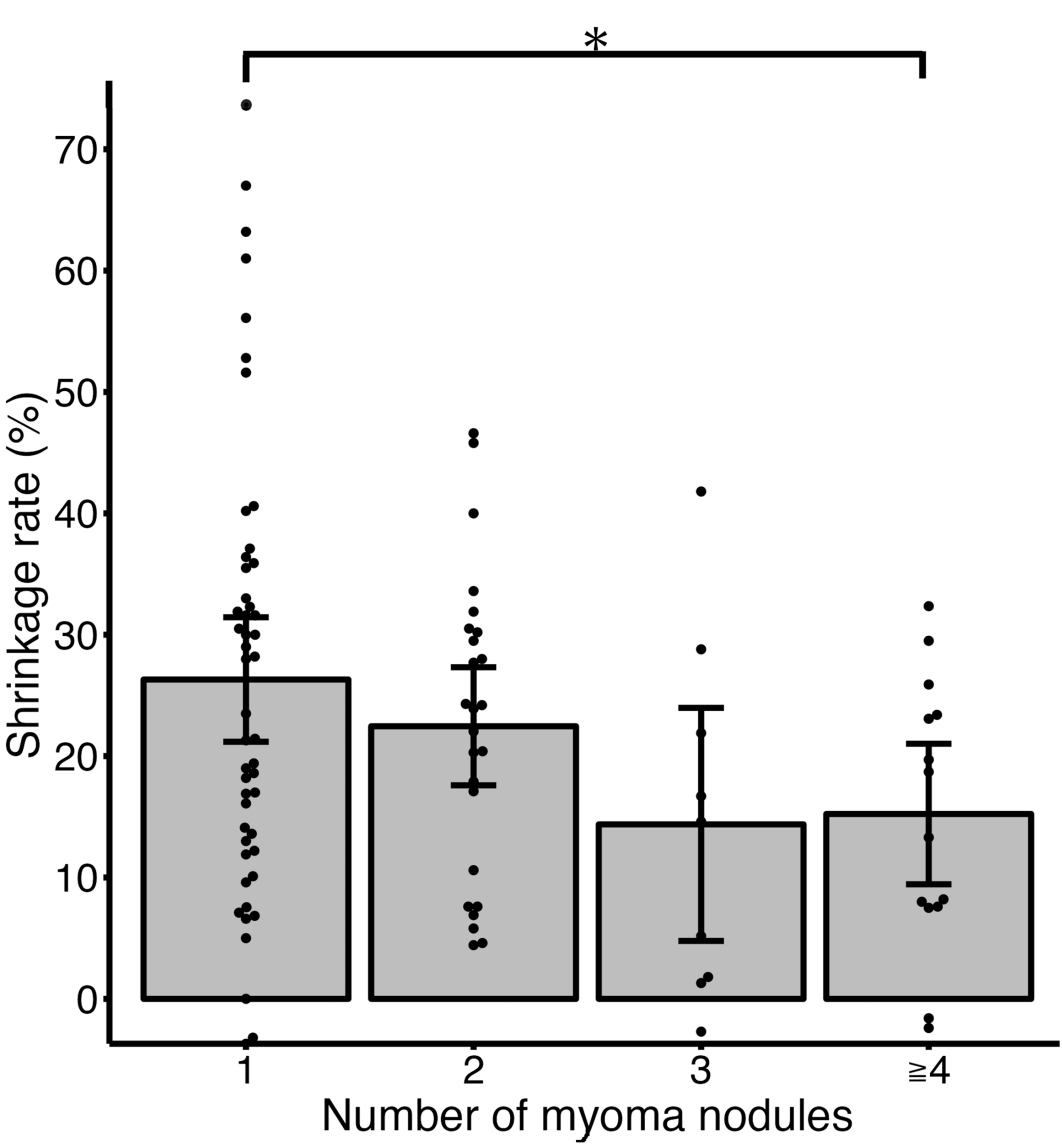
Mean shrinkage rate at 10-year after menopause with 95% confidence interval based on numbers of myoma nodules. The group with a single nodule showed a significantly greater shrinkage rate compared to the group with four nodules (26.3% vs 15.2%). Dots show each patient’s shrinkage rate. Bars show 95% confidence interval. The asterisk shows a p-value of 0.036.

**Figure 3:**
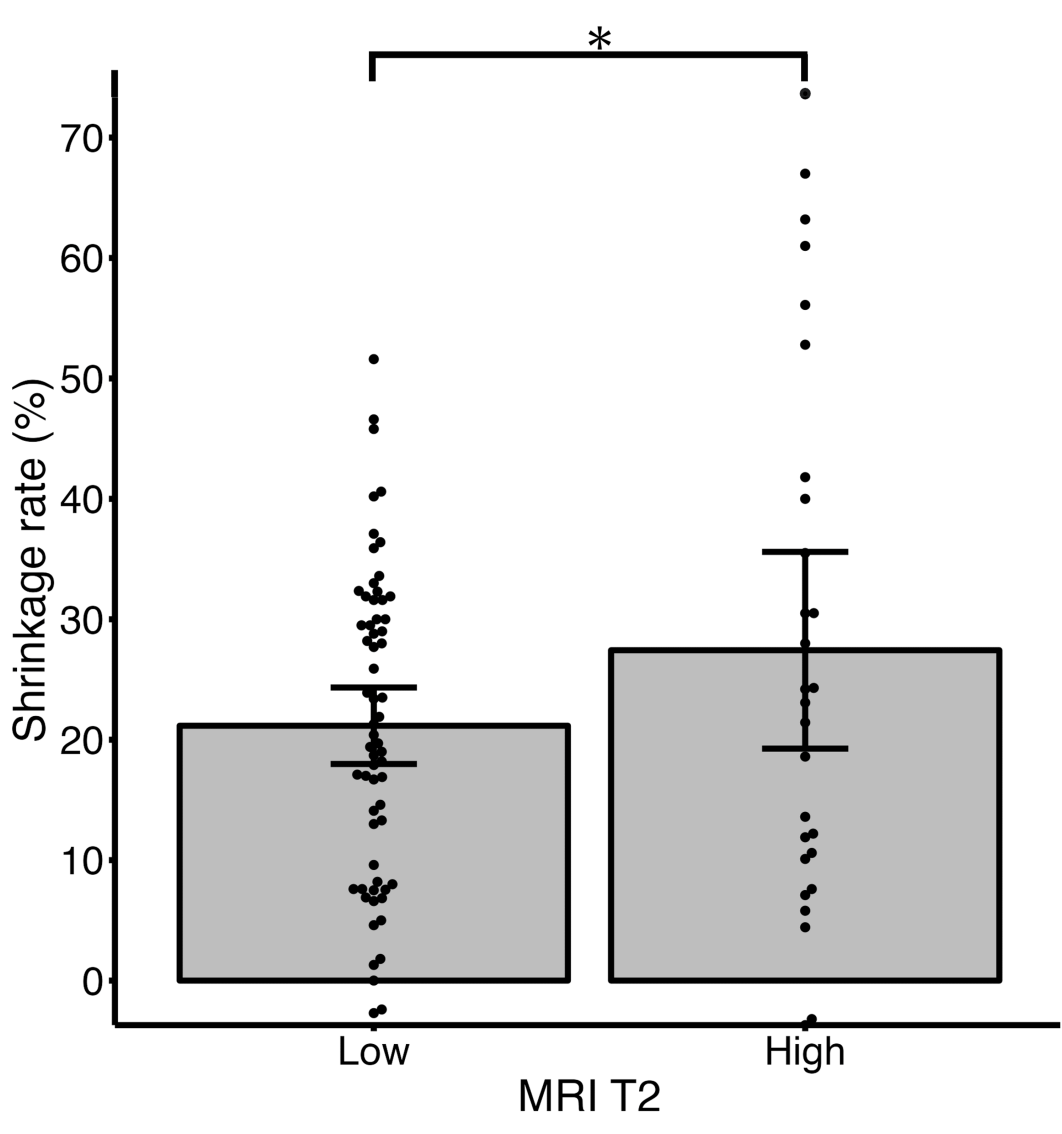
Mean shrinkage rate at 10-year after menopause with 95% confidence interval based on signals of MRI T2-weighted image. The group with high signal intensity on MRI T2-weighted images tended to have a greater shrinkage rate compared to the group without high signal intensity, although this difference was not statistically significant (27.4% vs 21.2%). Dots show each patient’s shrinkage rate. Bars show a 95% confidence interval. The asterisk shows a p-value of 0.092. Low indicates the group without high signal intensity on MRI T2-weighted images. High indicates the group with high signal intensity on MRI T2-weighted images.

In the examination of 10-year shrinkage rate, the group with a BMI of less than 25 showed a significantly greater shrinkage rate compared to the group with a BMI of 25 or more (25.0% vs 15.7%, p=0.023) (Figure 1). Additionally, the group with one nodule showed a significantly greater 10-year shrinkage rate compared to the group with four or more nodules (26.3% vs 15.2%, p=0.036) (Figure 2). The group with high signal intensity on MRI T2-weighted images tended to have a greater 10-year shrinkage rate compared to the group without high signal intensity, although this difference was not statistically significant (27.4% vs 21.2%, p=0.092) (Figure 3). When examined by parity and the location of myomas, no significant differences were observed (the corresponding figure or table is not presented). Furthermore, there was no significant correlation between the maximum diameter of myomas before menopause or the age at menopause and 10-year shrinkage rate (the corresponding figure or table is not presented).

**Figure 4:**
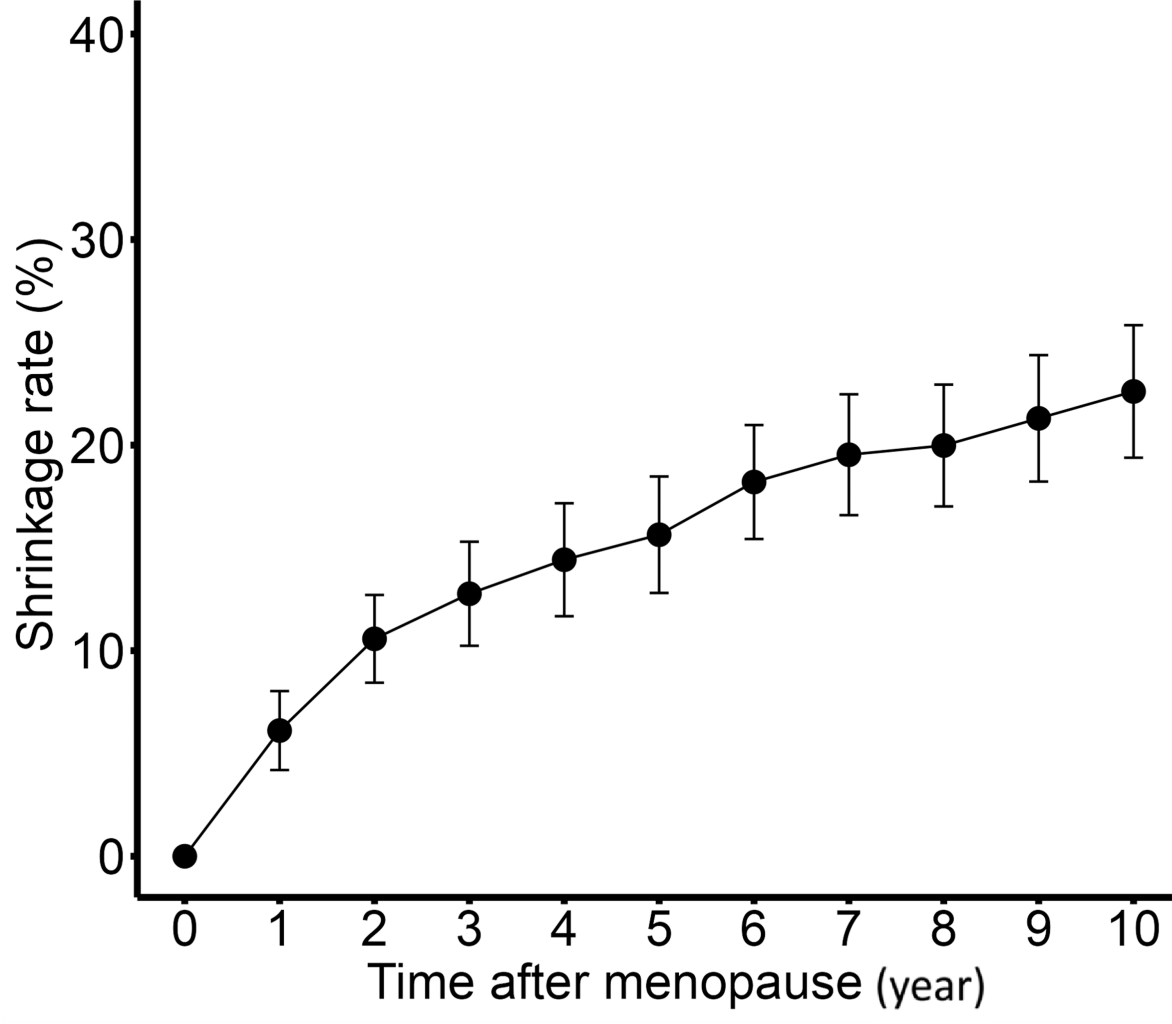
Annual trend of the shrinkage rate up to 10 years after menopause of 97 cases. The annual trend up to two years after menopause was significantly faster compared to the trend from the third to the 10th year in all patients (difference in slope: 3.888 points per year p<0.001). Bars show 95% confidence interval.

Stepwise multiple linear regression analysis showed that BMI (<25 or ≥25) (β= -9.053, p=0.020) and number of nodules (β = -4.467, p=0.003) significantly affected the 10-year shrinkage rate (Table 2). For annual trends, the annual trend up to two years after menopause was significantly faster compared to the trend from the third to the 10th year in all patients (difference in slope: 3.888 points per year, p<0.0001) (Figure 4).

**Table 2:** Stepwise multiple linear regression analysis of factors affecting the shrinkage rate 10 years postmenopause. BMI and the number of nodules significantly affected the shrinkage rate at 10 years postmenopause. BMI is categorized as <25 or ≥25. Beta (β) indicates partial regression coefficient. R^2^=0.1617; adjusted R^2^=0.1431

Variables	β	95% CI
Intercept	33.495	26.965 to 40.024
BMI	-9.053	-16.666 to -1.441
Number of myoma nodules	-4.467	-7.352 to -1.582

**Figure 5:**
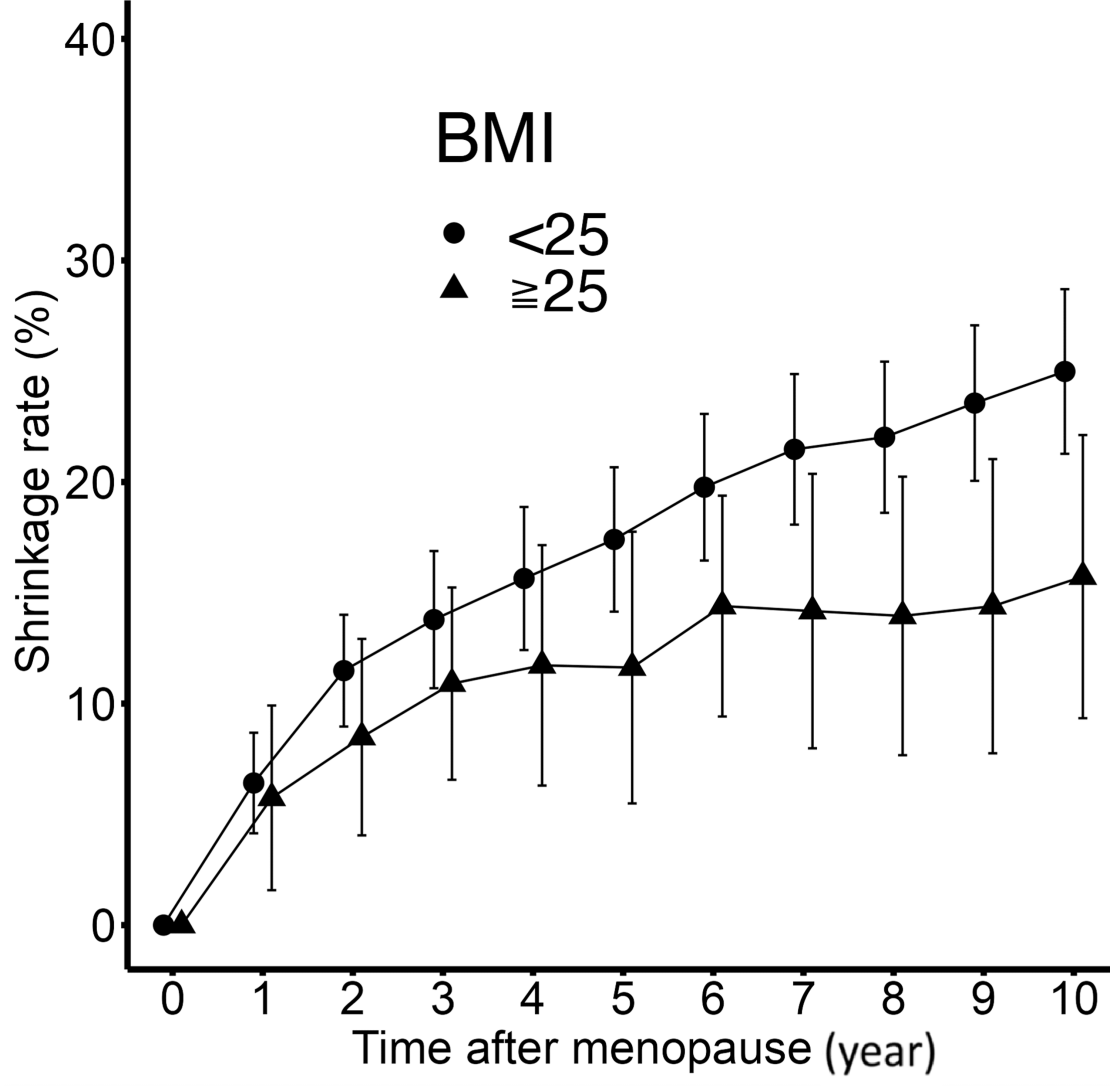
Annual trend of the shrinkage rate based on BMI up to 10 years after menopause. BMI <25 group and BMI ≥25 group showed that the annual trends up to two years after menopause were significantly faster compared to the trends from the third to the 10th year (difference in slope: 4.1531 points per year, p<0.001, and difference in slope: 3.601 points per year, p=0.001, respectively). Bars show 95% confidence interval.

Additionally, when divided into two groups based on BMI (less than 25 and 25 or more), both groups showed that the annual trends up to two years after menopause were significantly faster compared to the trends from the third to the 10th year (difference in slope: 4.1531 points per year, p<0.0001, and difference in slope: 3.601 points per year, p=0.0001, respectively) (Figure 5).

**Figure 6:**
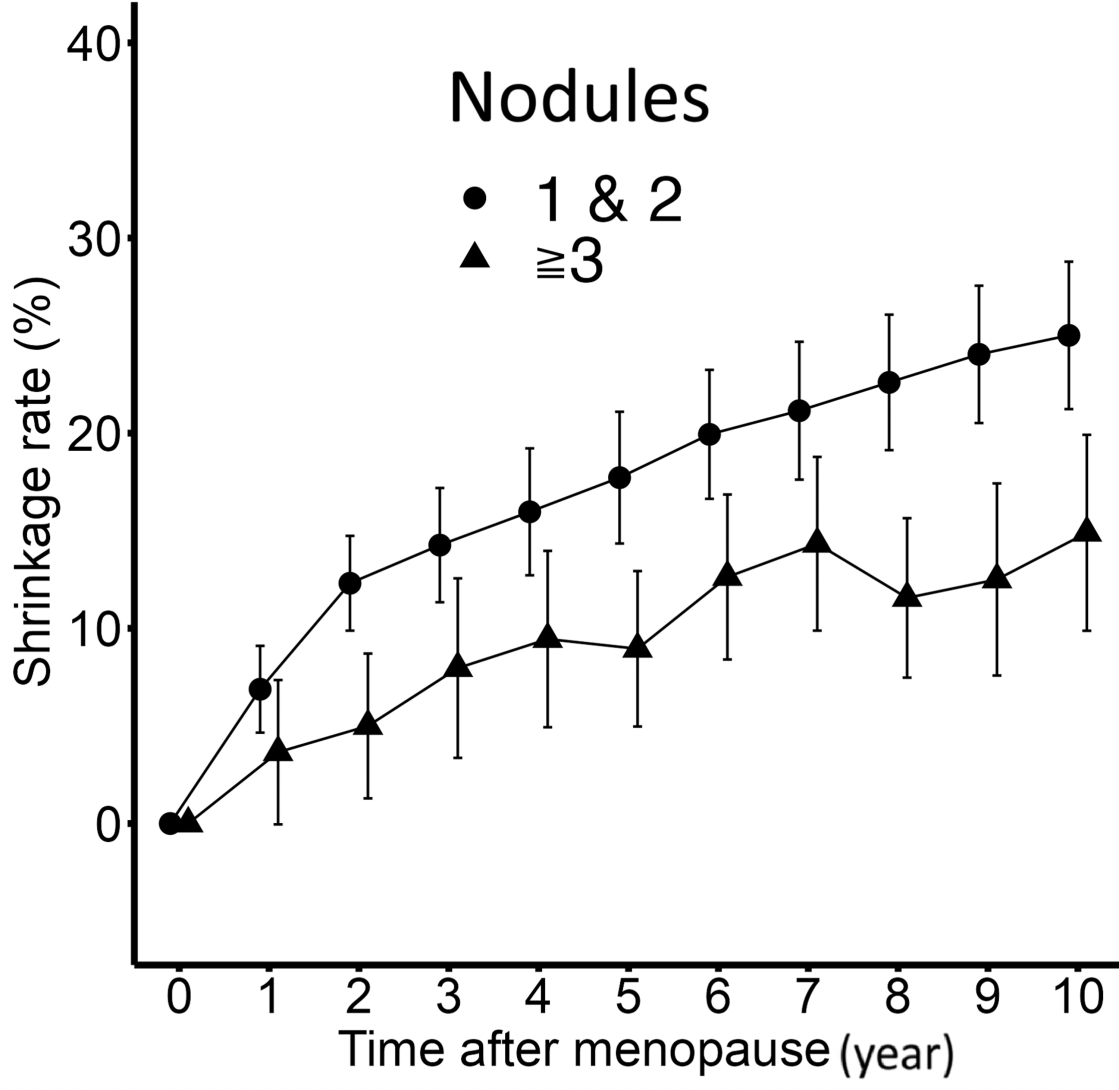
Annual trend of the shrinkage rate based on the number of nodules up to 10 years after menopause. The group with one or two nodules showed a significant difference in the annual trend between up to two years after menopause and from the third to the 10th year (difference in slope: 4.5904 points per year, p<0.001), but the group with three or more nodules did not show a significant difference in the annual trend between up to two years after menopause and from the third to the 10th year (difference in slope: 1.6259 points per year, p=0.107). Bars show 95% confidence interval.

On the other hand, when divided into two groups based on the number of nodules (one or two, and three or more), the group with one or two nodules showed a significant difference in the annual trend between up to two years after menopause and from the third to the 10th year (difference in slope: 4.590 points per year, p<0.0001), but the group with three or more nodules did not show a significant difference in the annual trend between up to two years after menopause and from the third to the 10th year (difference in slope: 1.626 points per year, p=0.107) (Figure 6).

## Discussion

It is generally said that uterine myomas shrink after menopause, and there have been previous reports indicating the shrinkage of myomas after menopause [2-4]. One of the few studies conducted by our group previously followed patients for up to five years [3]. In this previous report, we collected premenopausal myoma tissue with needle biopsy and found that myomas with high estrogen receptor expression, as determined by immunostaining for hormone receptors, tended to shrink more easily after menopause. Although this study was considered innovative, needle biopsy is not a test conducted at all facilities. Additionally, the observation period of two years (1.5-5 years) was not particularly long. Therefore, we considered that long-term observational studies are necessary to obtain information that will be useful in daily clinical practice. In this study, we aimed to analyze the clinical factors related to shrinkage of myomas in cases observed over 10 years after menopause and to elucidate the factors influencing the shrinkage rate after 10 years of menopause as well as the annual trend of shrinkage. This research is unique in that it involves 10-year follow-up period, and no similar studies have been reported to date. In this study, we targeted uterine myomas with a maximum nodule diameter between 50 mm and 160 mm. This criterion was based on our previous study where we validated the accuracy of myoma long diameter measurements using MRI and transabdominal ultrasound for nodules between 50 mm and 160 mm [5]. High signal nodules on MRI are reported to show a significant reduction effect with pseudomenopausal therapy [6]. Although this tendency was observed in our study, it was not statistically significant, possibly due to the small number of cases with high signal areas and the differences between natural and iatrogenic menopause. From the results of this study, it was found that cases with BMI of less than 25 and/or fewer nodules had a greater shrinkage rate 10 years after menopause. In cases with high BMI, the possibility that non-ovarian estrogen impacts the shrinkage rate was considered. It is known that in postmenopausal females, there is a positive correlation between BMI and blood estrogen levels [7]. This phenomenon is believed to be due to the synthesis of estrogen by aromatase in adipocytes [8]. So, it is believed that the decrease in estrogen due to menopause is more gradual in cases with high BMI, resulting in a slower rate of myoma shrinkage. Regarding the relationship between number of nodules and shrinkage rate, there are conflicting reports on the effect of number of nodules on the efficacy of UAE. One study reported that number of nodules affects UAE outcomes [9], while another reported that the number of nodules does not affect UAE outcomes [10]. It is anticipated that a uterus with multiple large myoma nodules may have a poor environment for growth, leading to a higher likelihood of hyalinization due to chronic blood flow insufficiency. There are reports that hyalinized uterine myomas respond poorly to pseudomenopause therapy and UAE, suggesting that they may also be resistant to natural shrinkage [11]. No previous studies have examined the relationship between the number of myoma nodes and spontaneous shrinkage after menopause, so conclusions are left to our speculation. When examining the annual trends in shrinkage rate, the shrinkage during the first two years after menopause was significantly faster than in the subsequent years. It is believed that uterine myomas experience rapid shrinkage due to the simultaneous effects of hormonal decline and reduced blood flow in the early postmenopausal period. Such changes are observed up to the second year, followed by a relatively gradual shrinkage. However, cases with three or more nodules showed a consistent shrinkage speed, possibly because these cases are chronically under poor blood flow and can adapt to the adverse changes of menopause. There is no prior research on this either and it is a matter of speculation.

This study has several limitations due to its retrospective nature. Firstly, changes in BMI during the study period were not adjusted. Secondly, despite being based on the results of previous study, there is a certain margin of error in the measurements obtained by transabdominal ultrasonography. Lastly, myomas with chromosomal abnormalities were not excluded. Nevertheless, this study, examining the long-term shrinkage of uterine myomas over a 10-year period after menopause, provides valuable information for both patients and gynecologists in managing uterine myomas.

## Conclusions

When following up on uterine myomas, both patients and gynecologists are interested in how much and over what period these myomas naturally shrink after menopause. In this study, we investigated the natural shrinkage of uterine myomas over a long-term period of 10 years after menopause, using annual transabdominal ultrasonography. The results indicated that BMI and number of myoma nodules were significantly related to the shrinkage rate of myomas over the 10-year period after menopause. Additionally, we found that myomas shrank significantly faster within the first two years after menopause compared to the later period. However, when there were multiple nodules with a maximum diameter of 50 mm or more, the early shrinkage rate did not differ significantly from the rate in the later period, suggesting that multiple myomas affect the early shrinkage rate after menopause.
